# Sex Differences in VO_2max_ and the Impact on Endurance-Exercise Performance

**DOI:** 10.3390/ijerph19094946

**Published:** 2022-04-19

**Authors:** Kelsey J. Santisteban, Andrew T. Lovering, John R. Halliwill, Christopher T. Minson

**Affiliations:** Department of Human Physiology, University of Oregon, Eugene, OR 97403, USA; ksantist@uoregon.edu (K.J.S.); lovering@uoregon.edu (A.T.L.); halliwil@uoregon.edu (J.R.H.)

**Keywords:** sex differences, oxygen consumption, athletic performance, exercise physiology

## Abstract

It was not until 1984 that women were permitted to compete in the Olympic marathon. Today, more women than men participate in road racing in all distances except the marathon where participation is near equal. From the period of 1985 to 2004, the women’s marathon record improved at a rate three times greater than men’s. This has led many to question whether women are capable of surpassing men despite the fact that there remains a 10–12% performance gap in all distance events. The progressive developments in sports performance research and training, beginning with A.V. Hill’s establishment of the concept of VO_2max_, have allowed endurance athletes to continue performance feats previously thought to be impossible. However, even today women are significantly underrepresented in sports performance research. By focusing more research on the female physiology and sex differences between men and women, we can better define how women differ from men in adapting to training and potentially use this information to improve endurance-exercise performance in women. The male advantage in endurance-exercise performance has commonly been attributed to their higher VO_2max_, even when expressed as mL/kg/min. It is widely known that oxygen delivery is the primary limiting factor in elite athletes when it comes to improving VO_2max_, but little research has explored the sex differences in oxygen delivery. Thus, the purpose of this review is to highlight what is known about the sex differences in the physiological factors contributing to VO_2max_, more specifically oxygen delivery, and the impacts on performance.

## 1. Introduction

**Figure 1:**
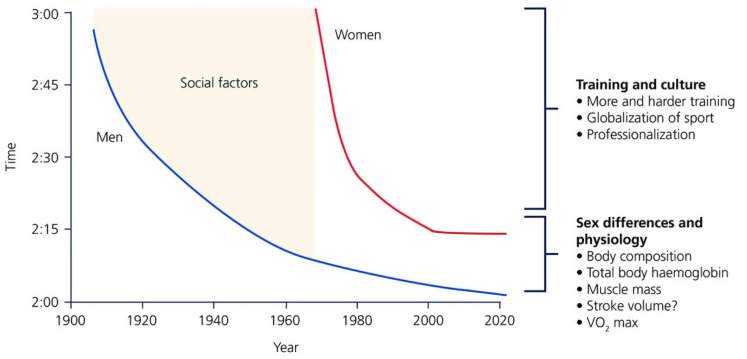
Reprinted with permission from Ref. [3]. 2017 The Physiological Society. History of world records in the marathon for men and women, modified slightly to show current world record times. The impact of changes in training and culture has helped with the steep improvement in women’s marathon record times through 2005. Physiological sex differences are primarily responsible for the gap that still exists between men’s and women’s times in all distances of individual endurance events, although cultural and training differences still exist.

When the modern Olympics were established in 1896, no women were allowed to compete in any event. It wasn’t until almost 100 years later, in 1984, that women were first allowed to compete in the Olympic marathon, still 12 years after first gaining legal allowance to compete in the Boston and New York marathons. Since then, female participation in the marathon, along with many other endurance events, has increased substantially. In fact, 61% of all road race registrants in 2019 were female, and nearly 50% of participants in marathon races are now women [1]. With increased participation has come marked improvements in performance, as women began to train at volumes and intensities comparable to men. Between 1985 and 2004, women’s marathon performance improved at nearly three times the rate of men, leading many to wonder if women would soon catch up to, or even outperform men [2]. That being said, a 13-min gap in world record marathon time still exists between men and women, and across all distance events, there is a consistent gap of ~10–12% between men and women [2,3] (Figure 1). This suggests that men continue to have a physiological advantage when it comes to endurance sports performance.

Since the 1920s, the field of exercise physiology has grown dramatically, starting with A.V. Hill’s interest in the study of athletic performance. It was Hill’s introduction of the concept of maximal oxygen consumption (VO_2max_) and its impact on endurance-exercise performance that has allowed modern researchers to build an extensive conceptual framework explaining the limits of human performance [4]. The progressive developments in sports performance research over the last 100 years have allowed elite athletes to continue to achieve athletic feats beyond what was originally thought possible. However, until recently, most research studies have utilized only male subjects, leaving female athletes largely under-represented in sport and exercise research. In his 2017 review exploring the physiological limitations of female endurance athletes, Dr. Michael Joyner expressed that, “for almost all issues outlined in this paper, there are either fewer or much fewer data on elite women vs. elite men” [3]. Similarly, Costello et al. in 2014 reported that women only made up roughly 35% of research subjects in performance studies [5]. Until the 1980s, it was widely assumed that physiological responses to exercise did not truly differ between men and women [6]. Most current training practices are based on research primarily conducted on men; thus, female athletes are expected to respond like men, creating training standards likely to be unreflective of female athletes’ needs [7]. By focusing more research on the female physiology and sex differences between men and women, we can better define how women differ from men in adapting to training and use that information to better inform female training methodologies, and potentially increase female endurance-exercise performance. Fortunately, in recent years, increased attention has been placed on the lack of equality between men and women in many areas of society, including research. Over the past 6 years, research initiatives have aimed to include more female subjects in clinical trial research and emphasize physiological differences between men and women [8]. These are important steps, but significantly more research specific to women is warranted.

### 1.1. Maximal Oxygen Consumption (VO_2max_)

The male advantage in endurance-exercise performance has been primarily attributed to the sex difference observed in VO_2max_ [4], a key determinant of aerobic performance. Even truly elite women have VO_2max_ values ~10% lower than those seen in men of similar elite status when expressed as mL/kg/min [4]. Whole-body oxygen consumption (VO_2_) increases with increasing exercise intensity as a consequence of greater oxygen demand from the exercising muscles. This increase in oxygen demand occurs because almost all the energy (i.e., ATP) utilized during an endurance-exercise performance (aerobic exercise) will be resynthesized in the mitochondria through oxidative metabolism [9]. With this rise in oxygen consumption, concurrent increases in a number of cardiovascular and respiratory variables occur such as heart rate, ventilation, and stroke volume. Essentially, we can think of VO_2_ as the ability for oxygen delivery to, and utilization by, the muscles during exercise. As intensity approaches maximal levels, VO_2_ reaches a plateau unaffected by further increases in exercise intensity [10]; this plateau is termed VO_2max_. VO_2max_ represents the maximal rate at which ATP can be resynthesized through aerobic pathways and creates a limitation to exercise tolerance [11].

VO_2max_ is determined by the product of maximal cardiac output, which can be broken down into stroke volume times heart rate, and the maximal arterio-venous O_2_ content difference (∆a-vO_2_). The limitation of oxygen delivery to the exercising muscles by “central” hemodynamic factors is most commonly considered the limiting factor in elite athletes to further increases in VO_2_ at extremely high exercise intensities [12]. The central factors involved in O_2_ delivery during exercise include pulmonary ventilation, diffusion across the pulmonary capillary membrane, cardiac output, and hemoglobin mass, in addition to peripheral factors such as skeletal muscle blood flow, and diffusion of O_2_ from the microcirculation into the muscle [9]. Elite female athletes’ inability to match the high oxygen consumption of their male counterparts is often attributed to those central factors—women typically have smaller hearts, lungs, and lower hemoglobin mass than men—limiting their capacity to deliver oxygen to the working muscles.

### 1.2. Other Determinants of Endurance-Exercise Performance

VO_2max_ alone cannot discriminate the performance capabilities in groups of endurance athletes that have similarly high VO_2max_ values [11]. Rather, there are a number of other physiological factors that impact the inter-individual variability between elite endurance athletes. These include running economy, lactate threshold, and critical power, and combined help to determine the average speed that an athlete can sustain during a distance event [11]. Which factors have the biggest impact in any given distance of race will depend on many factors, including pacing strategies.

#### 1.2.1. Running Economy

Running economy describes the oxygen cost of running at a certain speed or distance and can vary by as much as 30–40% amongst elite individuals [11,13]. The basis of these inter-individual differences in running economy remains somewhat elusive to researchers [14] but are known to be impacted by both physiological and anthropometric factors such as a high type I skeletal muscle proportion, increased mitochondrial volume, biomechanical factors, and breathing economy [15]. An athlete with a good running economy can utilize a lower percentage of their VO_2max_ for a given running velocity, reducing both glycogen utilization and reliance on anaerobic metabolism during an endurance competition [11]. While VO_2max_ has been shown to remain fairly consistent over time in elite athletes, the running economy will often improve throughout their athletic careers [11]. When using anthropometric scaling to body mass, recent studies have shown that women have a better running economy compared to men [16,17]. Mendonca et al. showed that when comparing men and women with similar percent differences from predicted VO_2max_, women showed better running economy consistently across a broad spectrum of submaximal running speeds [16]. Despite a 25% difference in VO_2max_ between the sexes in this study, maximal aerobic speed was only 18% different, indicating that running economy could partially compensate for women’s reduced VO_2max_ [16]. Stoa et al. also showed that, when scaled for body weight, women showed a 9% better oxygen cost of running compared to men. This study included long-distance runners with performance levels ranging from elite to regional [17].

#### 1.2.2. Lactate Threshold

The lactate threshold represents the highest intensity of exercise that can be performed before lactate removal exceeds lactate production, resulting in blood lactate accumulation during exercise. Exercise near the lactate threshold can be sustained for >2 h with blood lactate concentrations remaining stable and only slightly elevated [11]. The lactate threshold has been shown to improve with endurance training, with these improvements attributed to increased muscle lactate transport capacity and a higher proportion of type I skeletal muscle fibers, which both slow the accumulation of lactate in the blood [15,18]. The lactate threshold is often expressed as a fraction of VO_2max_ [11], and elite athletes are capable of sustaining 80–90% of their VO_2max_ for long-duration exercise with only slight increases in blood lactate [4]. The lactate threshold has previously been shown to be highly correlated with performance in distance running events such as the marathon [4], such that for two hypothetical athletes with a similar VO_2max_ and similar running economy, the athlete with the lactate threshold closer to their VO_2max_ would be expected to perform better. Along these lines, Stoa et al. [17] found slightly higher lactate thresholds in women compared to men (85% vs. 83% of VO_2max_). However, the authors noted that with higher VO_2max_ in the men, women still had lower running velocities at the lactate threshold [17]. (They observed a similar pattern of higher VO_2max_ but similar lactate threshold resulting in higher running velocities at lactate threshold when comparing the elite, national, and recreational athletes.)

#### 1.2.3. Critical Power

While the concept of the lactate threshold has been considered by exercise physiologists since the late 1970s and has significant time to work its way into the coaching vernacular, critical power has more recently emerged as a robust physiological model. Critical power represents the greatest metabolic rate that results in “wholly oxidative” energy utilization [19]. In terms of intensity, critical power is located above the lactate threshold and denotes the threshold between heavy and severe intensity exercise. Heavy exercise represents exercise that can be sustained for long durations while severe intensity exercise is that in which exercise tolerance becomes predictably limited [19]. Heavy intensity exercise is characterized by elevated but steady levels of blood lactate concentrations and VO_2_; however, once the critical power threshold is crossed, blood lactate and VO_2_ steadily increase until the exercise is terminated. Severe intensity exercise is also characterized by pronounced reductions in phosphocreatine content and pH, and increased lactate and inorganic phosphate concentrations within the exercising muscle [20]. The ability to sustain a given submaximal power output during long-duration events of greater than 1–2 h will be determined by critical power [20,21,22,23,24] but will be epiphenomenally related to lactate threshold (at least under many exercise conditions).

During exercise tasks where whole-body oxygen delivery is not required, such as single-limb isometric contractions, the power-duration relationship has been shown to differ between men and women [25]. Women exhibit greater fatigue resistance due to a greater proportional area of type I fibers and capillary density in the knee extensors. Therefore, when completing tasks that are not dependent on the cardiopulmonary system, women show a greater capacity for oxidative metabolism and fatigue resistance [26]. However, when considering whole-body exercise, factors that influence oxygen transport, such as ventilation, cardiac output, and hemoglobin have a significant influence [26]. When comparing the power–duration relationship between men and women during cycling, Ansdell et al. found no differences in women compared to men for relative critical power (74% vs. 72% of maximal power) [25], but as in the case above for lactate threshold, this translated into a lower absolute critical power. Thus, it is likely that when considering whole-body exercise, the female advantage disappears.

Although these additional parameters play an important role in impacting endurance-exercise performance across elite athletes, VO_2max_ is thought to be the biggest factor impacting sex differences in performance. Thus, the purpose of this review is to explore the current research investigating sex differences in the physiological factors contributing to VO_2max_, specifically those that determine oxygen delivery, and their impacts on performance. We begin with the pulmonary/respiratory system and then further delve into the various components of the cardiovascular system, including heart structure and other hematological factors.

## 2. Pulmonary and Respiratory Considerations

During sea-level exercise, alveolar ventilation must be adjusted such that arterial oxygenation is sufficient to have enough oxygen delivered to the exercising muscles. Generally speaking, the pulmonary system does a sufficient job of maintaining PaO_2_, PaCO_2_, and pH at close to resting levels over a wide range of submaximal work rates [27]. Even in most well-trained individuals, there is only a small 2–3-fold increase in alveolar-arteriolar oxygen difference (a-ADO_2_) from rest to VO_2max_, indicating an adequate rate of O_2_ diffusion across the alveolar-capillary membrane and maintenance of PaO_2_ during high intensities of exercise [28]. However, many elite endurance athletes have exceeded the capabilities of their lungs through training [29]. The structural and functional properties of the lungs and airways do not change in response to repetitive physical training [29]. Thus, in these elite athletes, the metabolic requirements associated with high-intensity exercise demand high ventilation rates (~200 L/min) and pulmonary blood flows which can actually reach and exceed the functional capacity of the respiratory system. This eventually compromises arterial oxygenation and consequently limb O_2_ delivery [30,31,32,33]. However, there remains a large variability in the extent of oxyhemoglobin desaturation amongst athletes. There are many athletes who remain unaffected by exercise-induced arterial hypoxemia (EIAH) even at maximal exercise, whereas others experience a fall in PaO_2_ starting at submaximal exercise, and yet the reasoning for this remains elusive [28].

During exercise, the pulmonary diffusing capacity increases in order to meet the rising oxygen demand of the skeletal muscle [29]. The oxygen diffusing capacity of the lungs is influenced by: (1) the recruitment of the pulmonary capillaries during exercise and the ability of these capillaries to distend in order to accommodate greater volumes of blood, and (2) the membrane diffusion capacity, or the number of alveoli recruited in the lungs [30]. However, highly fit endurance athletes can generate extremely high cardiac outputs (up to 40 L/min) in response to increasing intensities of exercise. This increase in cardiac output reduces the transit time of red blood cells through the pulmonary capillaries, limiting the opportunity for complete gas exchange between the alveoli and the blood. As a result, some athletes can experience decreased PaO_2_ and increased a-ADO_2_ (>25 mmHg) leading to EIAH, which can be a limiting factor in some highly trained athletes. At arterial oxygen desaturations of >4–5%, limitations to peak aerobic power start to occur [34,35,36]. Beyond this, changes in arterial oxygen saturation and VO_2max_ occur linearly such that each further 1% reduction in arterial oxygen saturation causes a ~1–2% reduction in peak aerobic power [28]. Harms et al. showed that by preventing EIAH in athletes with VO_2max_ values ranging from 115% to 200% of predicted, VO_2max_ was increased. By maintaining arterial blood oxygen near resting normoxia levels, VO_2_ increased significantly for a fixed maximal work rate, emphasizing the importance of EIAH on altered O_2_ content and O_2_ delivery rather than the metabolic capacity of the locomotor muscles in limiting VO_2max_ [34].

**Figure 2:**
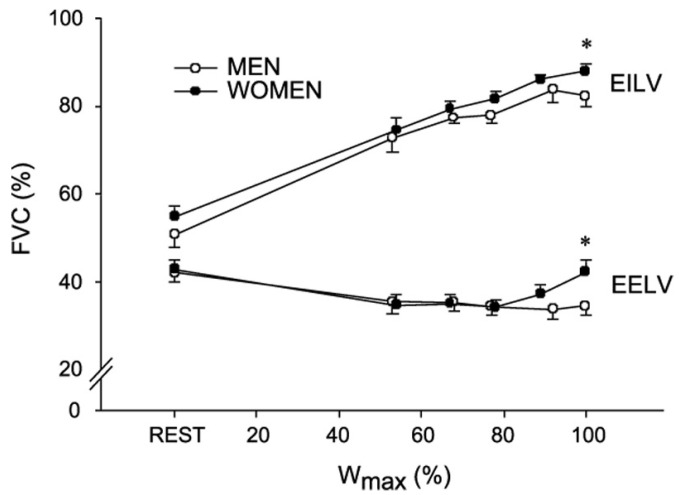
Reprinted with permission from ref. [41]. 2007 Blackwell Publishing. Regulation of lung volumes in men and women during progressive exercise to exhaustion. The end-expiratory (EELV) and end-inspiratory (EILV) lung volumes are expressed as a % of forced vital capacity (FVC). Higher EILV and EELVs were seen in women during progressive exercise, indicating dynamic hyperinflation. * Significantly different from men (*p* < 0.05).

Although reports are conflicting, women have been shown to exhibit EIAH at lower intensities when compared to men [31,34]. Women tend to have smaller lungs compared to height and age-matched men [37] and smaller airways when matched for height [38]. Due to decreased airway size, women must overcome greater resistive forces leading to the increased work of breathing for the given ventilation. Although this is only seen in significantly higher ventilations of at least 50–70 L/min, it can lead to a greater percentage of cardiac output that is redirected to the respiratory muscles to maintain adequate ventilation [39]. Furthermore, the smaller airway diameters and lung volumes result in lower peak expiratory flow rates and will reduce the capacity of females to increase ventilation compared to males. This may also predispose women to expiratory flow limitations earlier in exercise despite achieving lower ventilation than men [40,41,42,43,44]. The presence of an expiratory flow limitation may result in a relative hyper-inflation of the lungs to reduce expiratory flow limitation and allow for an increase in expiratory flow rate and allow for breathing at a larger lung volume [44]. However, breathing at a greater operating lung volume leads to an increase in the elastic work of breathing because lung compliance is reduced as lung volume increases to near total lung capacity. Collectively, this would redirect blood flow towards the respiratory muscles thereby reducing the amount of oxygen that is delivered to the working muscles and potentially reducing exercise performance by inducing fatigue at an earlier stage (see Figure 2) [44]. Interestingly, using heliox to minimize mechanical ventilatory constraints to exercise showed equal improvements in performance in men and women with no sex differences. This suggests that sex-based differences in lung anatomy and mechanics may not be great enough to affect performance [45]. In another study utilizing the negative pressure technique to assess expiratory flow limitation and work of breathing in a group of female endurance athletes during exercise, Guenette et al. found that nine of the ten women experienced significant expiratory flow limitation during maximal exercise. The lone subject who did not experience a flow limitation was also shown to have the lowest work of breathing and the greatest force vital capacity of all the subjects. In fact, this woman had a lung volume comparable to men who were significantly taller (5.4 L vs. 5.6 L), indicating that lung size, rather than sex per se, is the primary determinate of differences in respiratory function during exercise [41].

Women may have greater fatigue resistance in the skeletal muscle due to sex differences in muscle morphology, substrate utilization, and overall muscle mass [46]. Thus, it has also been suggested that women may experience less respiratory muscle fatigue when compared to men [42]. When exploring sex differences in the activation of the inspiratory muscle metaboreflex, Smith et al. found that women exhibited less of an increase in blood pressure and leg vascular resistance, as well as less of a decrease in leg blood flow compared to men [47]. Welch and colleagues went a step further by assessing diaphragmatic fatigue directly. They found that inspiratory muscle endurance time was significantly increased in women versus men and that at a similar degree of diaphragmatic fatigue, women showed less of an increase in heart rate and blood pressure [48]. Taken together, although women may show a greater work of breathing at a given ventilation, this may be combatted, at least in part, by a greater fatigue resistance of the respiratory muscles and aid in reducing or abolishing the limitations caused by increased work of breathing amongst female athletes.

**Figure 3:**
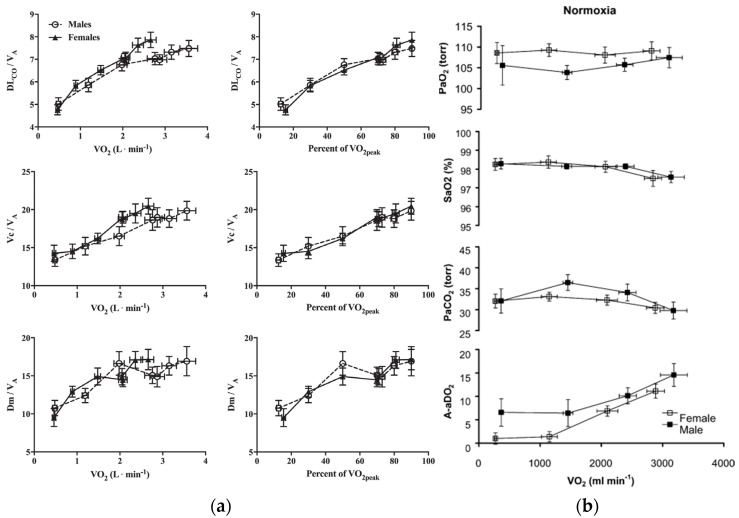
(**a**) Reprinted with permission from Ref. [49]. 2017 the American Physiological Society. Diffusion capacity, pulmonary blood volume, and membrane diffusing capacity responses to exercise, corrected for alveolar volume, in male and female subjects. No significant differences were found. (**b**) Reprinted with permission from Ref. [50]. 2004 The Physiological Society Arterial PO_2_, saturation, PCO_2_, and alveolar-arterial PO_2_ difference at rest and during exercise in normoxia. Male and female subjects are matched for age, height, and VO_2max_.

The anatomical differences seen between men and women may also indicate that women experience reductions in diffusion capacity compared to men due to a smaller surface area for gas exchange and a lower air flow rate. This can potentially cause women to be more susceptible to EIAH during situations of high oxygen demand [40]. When Bouswema et al. examined the diffusion capacity response to incremental exercise in height-matched, physically active men and women, it was found that women did exhibit consistently lower pulmonary diffusing capacity, capillary blood volume, and membrane diffusing capacity (Figure 3b). However, these sex differences were eliminated when the alveolar volume (or lung size) was accounted for [49]. Using a subgroup of highly fit men and women, they saw no difference in responses from rest to peak exercise leading them to conclude that there were no apparent sex differences that would contribute to reduced gas exchange in women [49]. A study conducted by Olfert et al. further confirms these findings as they found no differences in V/Q mismatch or diffusion limitation when comparing height, age, fitness, and lung volume matched men and women [50] (Figure 3a). Taken together, the preponderance of evidence suggests that any differences in pulmonary diffusion capacity during exercise may be attributed to lung size rather than actual sex differences, and that elite women are likely not at any severe disadvantage when compared to elite men. Additional studies considering the independent effects of body size and lung size on exercise are warranted.

## 3. Cardiac Considerations

Unlike untrained individuals, endurance-trained athletes do not demonstrate a plateau in stroke volume during exercise [51,52,53,54]. The increased volume load placed on the heart during exercise increases stroke volume in endurance athletes, and therefore cardiac output and VO_2_ are greater at peak exercise in these athletes compared to less fit individuals [54]. Physiological cardiac hypertrophy (PCH) is considered to be a beneficial adaptation to chronic physical training that stems from an elevated demand for oxygen. The increased oxygen demand was met by a simultaneous increase in pulmonary VO_2_ and augmented cardiac output [55]. PCH can be characterized by an increased left ventricular mass, an enlargement of ventricular chamber size, and an overall improved cardiac function during exercise [55,56,57]. PCH is a common characteristic seen in endurance athletes: a 15–20% greater left ventricular wall thickness, and a 10% greater left ventricular cavity size have been reported in athletes compared to sedentary individuals [58]. This increase in cardiac mass can, in part, provide an explanation for these individuals’ ability to augment stroke volume and cardiac output in response to high levels of oxygen demand. Athletes’ hearts tend to have greater chamber compliance and distensibility than non-athletes, allowing for greater diastolic filling and increased ejection [15,59].

During high-intensity exercise, women reach lower maximal stroke volumes compared to men, resulting in a lower maximal cardiac output as maximal heart rate is similar across sexes. This is often attributed to the smaller heart and body size of women compared to men, even despite prolonged physical training. A number of studies have examined heart dimensions across large pools of elite athletes. In a seminal study conducted by Pelliccia et al., the hearts of 947 Italian athletes (~22% female) were measured via echocardiography in order to characterize the upper limits of exercise-induced hypertrophy across these subjects [60]. They found that males who participated in rowing, canoeing, or cycling have substantially greater left ventricular wall thickness than any of the other athletes, reaching up to 16 mm in diameter. The females in this study, regardless of sport, never exceeded a left ventricular wall thickness of 11 mm. In 2004, Whyte et al. conducted a similar study in order to define the upper normal limits of physiological hypertrophy in a large cohort of male and female British athletes. In this cohort, women comprised around 30% of subjects (*n* = 136) [61]. Similar to Pelliccia’s findings, no female athletes showed a left ventricular wall thickness of greater than 11 mm; however, a wall thickness of up to only 14 mm was observed in the male subjects. The authors expressed that although the women in the study had a qualitatively similar response to training, they had lower absolute cardiac dimensions compared to men.

**Figure 4:**
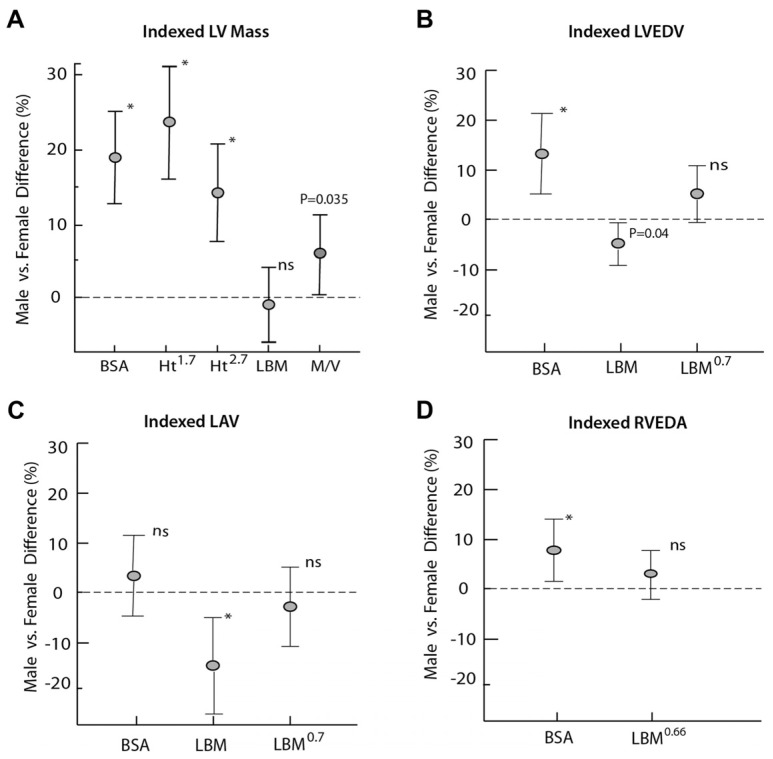
Reprinted with permission from ref. [62]. 2015 Elsevier Inc. Gender-associated relative difference in structural indexes using different scaling parameters. Allometric indexing to LBM significantly reduced differences between men and women. Data are presented for (**A**) LV mass, (**B**) LV end-diastolic volume, (**C**) left atrial volume, and (**D**) RV end-diastolic area. * significant difference between males and females (*p* < 0.05); ns is not significant.

One of the confounding factors when comparing absolute heart dimensions between men and women is body size, as men are typically larger than women and therefore have a greater overall heart size. Giraldeau et al. looked at ventricular dimensions in college athletes and sought to determine if sex differences persisted after normalizing for lean body mass [62] (Figure 4). They found that the gender-related differences in left ventricular mass tended to decrease significantly or disappear, leading them to conclude that differences in left ventricular mass appear to be, to a large extent, related to differences in body composition as women also have a higher percent of body fat compared to men. In their study, however, they only included athletes from low to moderate intensity, or non-endurance, sports. Pressler et al. conducted a similar study across a subject pool of over a thousand athletes across 40 different disciplines [59]. Although their findings were similar to those by Giraldeau et al., they found that in athletes from high intensity, or more endurance-driven sports, the differences between males and females remained significant despite scaling to lean body mass. This showed that the male athletes in particular exhibited exercise-induced cardiac adaptations beyond the sole influence of body composition [63].

**Figure 5:**
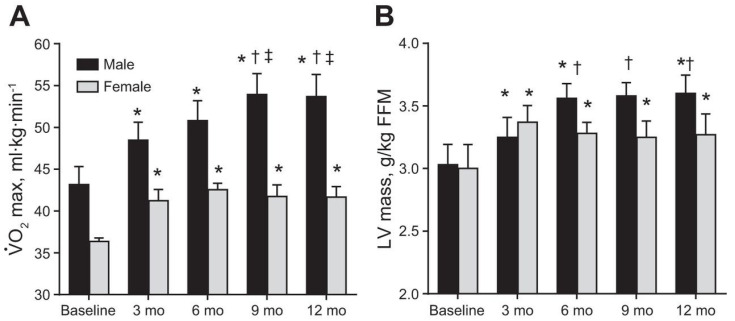
Reprinted with permission from ref. [64]. 2015 the American Physiological Society. (**A**) effect of 1 year of endurance training on VO_2max_ indexed to baseline body mass in males and females, (**B**) effect of 1 year of endurance training on changes in LV mass measured by MRI scaled to base-line fat-free mass. Post hoc comparison with baseline (*), with baseline, month 3 (†), and with month 6 (‡) for *p* < 0.05 from linear mixed model.

To explore sex differences in the development of PCH over time, Howden et al. conducted a study comparing the effects of one year of intensive training on previously sedentary individuals [64] (Figure 5). Through this study, a direct comparison between the physiological adaptations of the heart to endurance training was made between men and women. Using a similar training plan that matched for volume and intensity, they found that males had a more pronounced left ventricular hypertrophy throughout the one year of training. Females reached maximal left ventricular mass after only three months of training and did not show any further improvements. This increase in left ventricular mass allowed for greater augmentation of the Frank–Starling mechanism in men, allowing them to generate a greater stroke volume and therefore exhibit a greater cardiac output in response to an increase in filling pressure. These differences also corresponded to differences in VO_2max_, the women experienced a plateau after only three months of training, but the men saw increases in VO_2max_ until month nine of training.

The more progressive increase in male heart size has been thought to be due to the small elevations in androgens. One interesting observation from this study was that marked reductions in body fat were observed from month six onward in only female subjects. This occurred in response to an increase in training load. Thus, a possibility is that the lack of effect on VO_2max_ and cardiac mass could be due (in part) to a suboptimal energy intake in the female subjects, which could lead to impaired protein synthesis, limiting the amount of PCH these women experienced [64]. Female athletes are more likely than males to under-fuel throughout training [65], and therefore are more susceptible to injuries or blunted physiological adaptations related to low energy intake. As the participants were matched for volume and intensity, there is also the possibility that the training protocol better suited the needs of the male vs. female subjects.

This highlights the importance of developing training and nutrition standards made specifically for women, based on research on female subjects. In a review conducted by Diaz-Canestro and Montero [66], they found through a meta-analysis of eight training studies that endurance training consistently elicited greater increases in VO_2max_ in men compared with women. This trend held strong against potential moderating variables including subject characteristics and training methodology. Again, more studies are warranted to compare true sex differences in long-term endurance training adaptations. Only three of the eight studies investigated employed a training protocol for longer than eight weeks, and two of those three studies investigated older individuals (mean age = 64 years) with very low VO_2max_ values. It, therefore, remains unclear how endurance training adaptations differ between men and women long term.

## 4. Hematological Considerations

Oxygen transport within the blood is another limiting factor to achieving a higher VO_2max_ in athletes. Oxygen transportation in the blood is directly related to total hemoglobin mass as an increase in total hemoglobin mass by one gram leads to an absolute increase in VO_2max_ by 4 mL/min, and thus a normal to high hemoglobin mass is needed to reach a normal to high VO_2max_ [67,68,69]. Significantly greater values of hemoglobin mass have been observed in trained vs. non-trained subject and increases in hemoglobin mass have been reported as a physiological response to regular endurance training in untrained subjects [70,71]. However, training programs in previously untrained subjects are not sufficient to increase hemoglobin values to those reported for elite marathon runners [72], indicating that maximum total hemoglobin mass values are only reached through years of intensive training or are strongly influenced by genetic factors. When looking at oscillations in hemoglobin mass during a training year, there is very little change in these values even when comparing periods of recovery with high-level competitions [73].

**Figure 6:**
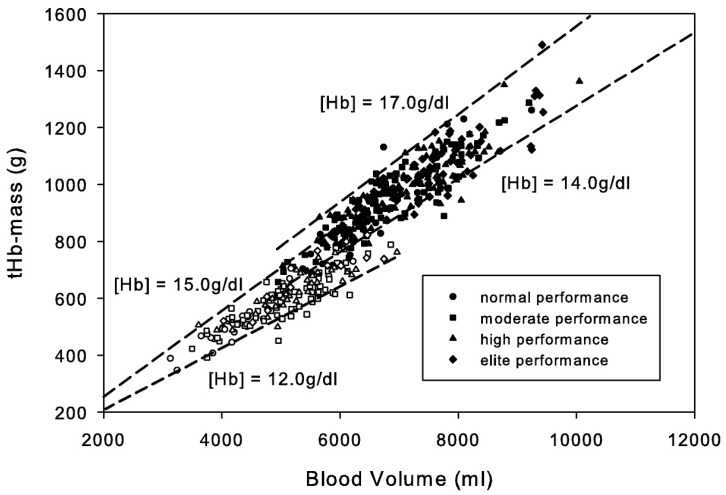
Reprinted with permission from ref. [69]. 2010 American College of Sports Medicine. Data are from 490 subjects (male *n* = 314, closed symbols; female *n* = 176, open symbols). Endurance state is classified by VO_2max_. The dashed lines indicate hemoglobin concentration as a function of blood volume and hemoglobin mass.

Independent of iron status, women have mean hemoglobin levels of 12% lower than age and race-matched men [69,74] (Figure 6). Therefore, for the same quantity of blood flow, women have an overall lower oxygen delivery. This is compounded by the fact that women also have a lower blood volume relative to body size, limiting how much blood flow can be redirected to the exercising muscles during exercise without compromising delivery to other tissues [58]. This difference in hemoglobin mass is not the result of circulating EPO levels, rather women seem to have a lower hemoglobin set point compared to men. This sex difference has been generally thought to be caused by a direct stimulatory effect of androgens in both the bone marrow and kidney in response to EPO, and an inhibitory effect of estrogen on the bone marrow in women [75,76].

Men, on average have 10–20 times higher levels of circulating testosterone compared to women [77]. However, in female athletes with hyperandrogenism, such as those with polycystic ovarian syndrome (PCOS), elevated levels of testosterone are seen [78]. In fact, women with PCOS tend to be over-represented in Olympic athletes as well as other athletic populations [77,79,80]. These athletes are anabolic with greater muscle mass and higher bone mineral density than other female athletes, allowing them greater injury resistance [78,79]. Further, endurance athletes with PCOS have been shown to have higher maximal oxygen uptake during treadmill exhaustion tests than other female athletes matched for BMI [78]. Androgen measures were shown to be positively correlated with VO_2max_ and lean mass in these athletes and could be influential in the higher athletic achievement of some athletes with PCOS [81]. Females with congenital adrenal hyperplasia (CAH), another condition where elevated androgen levels are present, have also been shown to have higher levels of hemoglobin and hematocrit [82], emphasizing the positive correlation between androgens and red blood cell mass. However, despite elevated levels of androgens in these females, men still have significantly higher levels of circulating androgens (Women: 0.1–1.8 nmol/L, Hyperandrogenic Women: 3.1–4.8 nmol/L, Men: 7.7–29.4 nmol/L) and consequently higher hemoglobin mass values.

**Figure 7:**
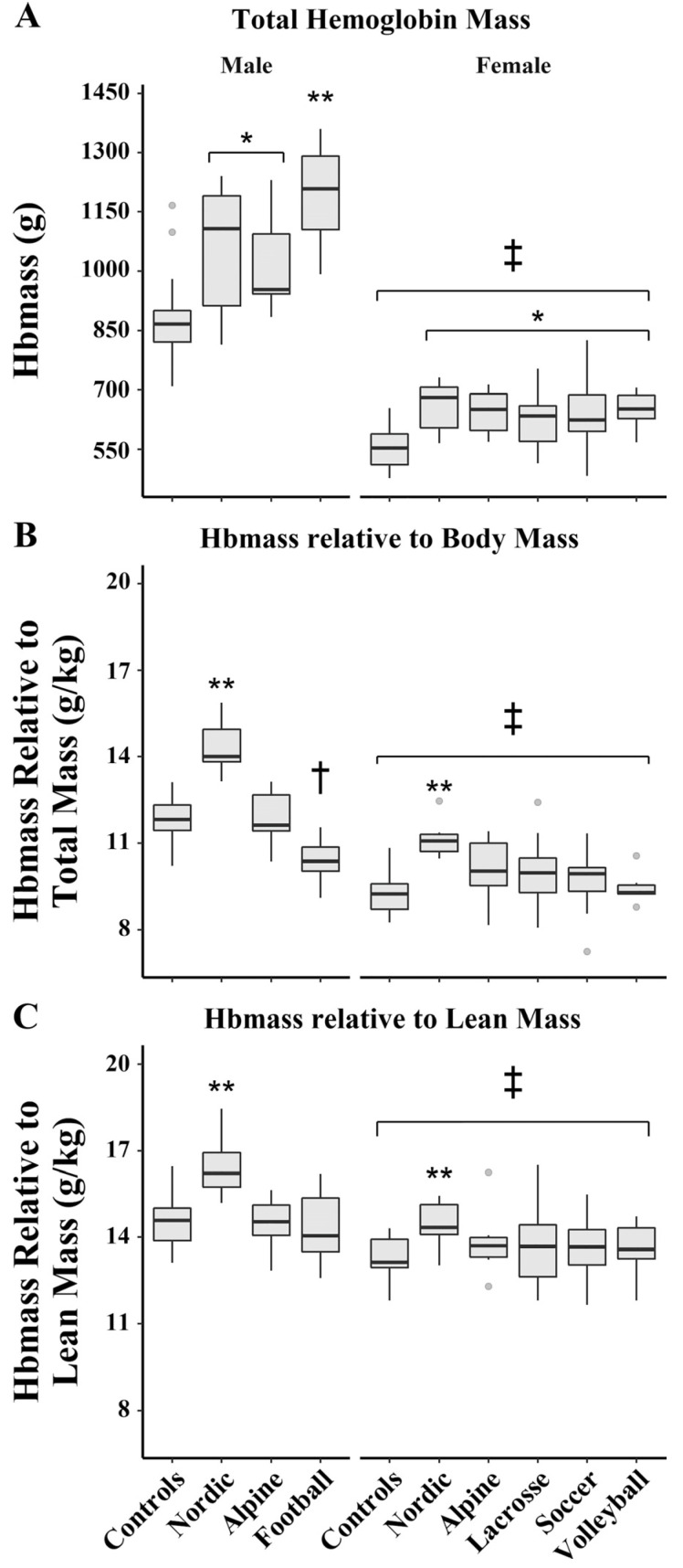
Reprinted with permission from ref. [86]. 2020 the American Physiological Society. Differences in total hemoglobin mass (**A**), hemoglobin mass relative to total body mass (**B**), and hemoglobin mass relative to lean mass (**C**). * Significantly higher than sex-matched control subjects; ** significantly higher than all other sex-matched groups; † significantly different from control and alpine; ‡ significantly lower than male subjects.

Lean body mass also plays an important role in determining hemoglobin mass [83,84,85,86] due to its high metabolic demand; an increase in lean mass requires a concomitant increase in hemoglobin mass in order to maintain adequate oxygen delivery. By normalizing hemoglobin mass by lean mass, Goodrich et al. explored the influence of athletic training, sex, and iron deficiency on hemoglobin mass [86]. When comparing sexes for absolute hemoglobin mass, males had 62.9% higher values compared to female athletes. However, when normalizing for lean body mass, the hemoglobin mass of male subjects was only 7.5% higher than female subjects, indicating a smaller sex difference in hemoglobin mass than has been observed previously (Figure 7).

In that study, females had over eight times higher odds of being iron deficient than male athletes, and showed a significant relationship between hemoglobin/lean body mass and ferritin, showing that iron deficiency was highly influential in determining hemoglobin mass in female subjects in particular. Generally, before puberty, no significant gender difference exists in hemoglobin, red blood cell count, and serum ferritin concentration. However, following the onset of menstruation in women, a significant difference emerges between sexes, which continues through menopause [87,88], and puts women at a higher risk for iron deficiency. Iron deficiency can play a role in decreasing erythropoiesis and hemoglobin mass through a decreased iron delivery to erythroid progenitor cells. This will impair oxygen delivery to the tissues and also lead to a deficiency of iron-containing compounds, namely enzymes, in various tissues [87,89]. Anemia may be highly influential on female athletes’ training as it has been shown to compromise athletic performance, despite a compensatory increase in cardiac output [90,91]. Iron deficiency impairs ATP production and increases reliance on the anaerobic metabolism of glucose to produce ATP [92]. Clearly, more research needs to be done on the impact of iron deficiency in female endurance athletes, and again more scientifically-backed nutritional guidelines need to be defined for women.

## 5. Training Strategies—Heat and Hydration

At the elite level, only small differences will separate the top endurance athletes. What is different across these athletes is their ability to train consistently, and beneficially adapt their training to best suit their performance needs. As mentioned previously, most established training methodologies are based on research primarily conducted on men. Research exploring sex differences in the adaptation to different training practices is severely limited.

From the 2020 Olympics in Tokyo to the 2021 Chicago marathon where temperatures peaked in the mid-80s Fahrenheit, endurance athletes are often subjected to high ambient conditions. Exercising for prolonged periods in hot conditions can greatly impact performance as the core temperature rises more quickly, leading to increased demands for heat dissipation. Heat dissipation occurs via two main methods (1) cutaneous vasodilation, and (2) increased sudomotor activity (sweating) [93]. In athletes not acclimated to hot weather, the increase in circulation towards the periphery competes with the demand for blood flow to the working muscles, eventually compromising performance.

Although current evidence shows no difference in the risk for heat-related illness between the sexes [94], many studies have shown differential responses to thermoregulation and heat acclimation in response to prolonged exercise in the heat. One main consideration for these differences is the impact of the menstrual cycle on thermoregulation in women. Although beyond the scope of this paper, one such example is the shift in the onset of sweating and cutaneous vasodilation to as much as a 0.6 °C higher core temperature during the luteal phase (high estrogen and high progesterone), potentially making women more susceptible to reaching hypothermic levels earlier in exercise during this period [95]. To explore more on changes in thermoregulation across the menstrual cycle, we direct you to the following review by Giersch and colleagues [95], but emphasize that this is a topic requiring further exploration, especially when defining training standards in the heat for female athletes.

Beyond the menstrual cycle, Gagnon et al. showed that when matching for heat generation and heat loss requirements, women have a lower sweating capacity for a given metabolic heat generation [96,97]. This has only been seen during higher intensity exercise, but may lead to decreased heat dissipation at higher workloads of exercise in the heat and may force women to compensate through increased peripheral vasodilation. This puts them at a greater risk of cardiovascular strain and orthostatic intolerance [98]. As discussed previously, female athletes have smaller hearts and lower stroke volumes when compared to male athletes, with increased vasodilation to the periphery there is a further reduction in circulation towards the working skeletal muscles. In fact, women ages 18–35 are already at five times higher risk of orthostatic intolerance compared to their male counterparts due to lower vascular sympathetic nerve activity [99,100] and increased beta-adrenergic vasodilation in the peripheral circulation [101,102]. This may largely impact elite female athletes as this is the age range at which they are usually most competitive. Taken together, women may be less efficient than men at thermoregulating during exercise in hot environments and have a greater requirement for heat acclimation training or precooling strategies, especially prior to competition in hot ambient temperatures.

Heat acclimation training improves heat transfer from the body’s core to the skin by reducing resting core temperatures, slowing the rise in core temperature, expanding plasma volume, reducing resting heart rate and rise, and increasing sweat rate at the onset of exercise in the heat [103,104]. It is recommended that both male and female athletes undergo heat acclimation prior to competition in particularly warm climates. Based on the sex differences observed in thermoregulation, it may be of higher importance to women prior to prolonged competition in the heat. Currently, recommendations for heat acclimation protocols are based primarily on research conducted on male athletes. However, best practices for heat acclimation need to be established independently for women as some research indicates that there may be differences in both mechanism and timeframe for acclimation [105]. One study conducted by Mee et al., compared men and women who underwent both a short-term heat acclimation protocol (STHA; 5 days) and a long-term heat acclimation protocol (LTHA; 10 days) to understand the potential differences in acclimation. Results showed that both males and females achieve partial adaptation following STHA but with males demonstrating a reduction in thermoregulatory and cardiovascular strain, and females demonstrating an increased sudomotor function. Contrary to the men, the women in the study did not see reductions in resting or peak core temperature after five days. This may indicate that women need a longer duration or an adjusted protocol for heat acclimation training prior to competition in the heat, and highlights the importance of updated guidelines for women [104].

Another important consideration for training and competing in the heat is hydration strategy, and again there has been little research conducted to delineate standards between men and women. Endurance-exercise performance is thought to be affected by dehydration via decreased blood volume associated with fluid loss during prolonged exercise [105]. The decrease in blood volume results in decreased stroke volume and an increase in heart rate for a given cardiac output resulting in greater cardiovascular strain for a given exercise workload. When combined with exercise in the heat this could diminish exercise capacity because more blood volume will be lost due to total body water loss from sweat [95,106]. Even mild dehydration of 1–3% body mass loss can have detrimental impacts on performance by increasing markers of thermoregulatory and cardiovascular strain [107,108].

The menstrual cycle alone has been implicated in altering fluid compartmentalization, fluid balance, and sudomotor function. However, there are currently no female-specific hydration guidelines and a serious lack of female-only studies focusing on hydration [108]. Women have a higher number & density of sweat glands but have been shown to sweat less than men for a given metabolic heat generation [96,108]. Although this may be indicative that women are less susceptible to dehydration than men, some research has shown that women experience greater thermoregulatory strain when exercising at a lower magnitude of dehydration [109,110]. The limited studies available have shown that when exercising at the same workload for the same time duration, the onset of the rise in core temperature occurs earlier in women compared with men. In some studies, this occurred at lower relative dehydration in women (0.5% vs. 1.5% body mass loss) [108,109,110,111,112]). Due to women’s smaller body size and higher adiposity than men, they possess a lower absolute total body water volume on average [113]. This suggests the absolute and relative body mass losses that occur during exercise-induced dehydration represent a larger portion of a female’s total body water [113], which may explain why women see a greater rise in core temperature at the same relative dehydration. This highlights the importance of sex-specific guidelines, as abiding by current hydration recommendations based solely on research on men could put women at a disadvantage. While they may have lower sweat rates and lower needs for hydration, they may have narrower margins for correcting hydration levels to avoid the negative consequences of dehydration.

## 6. Authors Closing Commentary

There is a large amount of research that still needs to be done on female athletes. Progress has been made, but much remains to be done. If we look back at Figure 1, Dr. Joyner attributes the steep improvement in marathon performance time in the last 40–50 years to cultural change. The “cultural change” he is referring to is women gaining allowance to train and compete in the same way men do. In the USA, Title IX had a huge impact on this. While significant, this may only mark the starting line for women in competition and performance research—the marathon course still lays ahead of us. We would propose that female athletes today are still being limited by cultural factors, but those factors largely stem from a lack of understanding of the female physiology on exercise performance and the application of data, derived from studies performed exclusively on men, to women. When this is done by either uninformed or misinformed coaches and athletic programs, it can lead to unfortunate outcomes such as overtraining, relative energy deficiency in sport (RED-S), and stress fractures or other training injuries. Beyond the physical, it can support the destructive culture of weight- and body-shaming which a number of athletes, both collegiate and professional, have been brought to light.

In this review, we have highlighted that there is much more work that needs to be done in female athletes. Performance research that has been conducted on men should not simply be applied to women, as it may place unrealistic expectations on these women athletes. As Stacy Sims has so eloquently stated, “Women are not small men” [114], and trying to reduce a woman’s body fat percentage to the value of a man’s will only cause her body to break down. Ignorance on this topic has created an unhealthy culture, especially for young athletes in high school and college who are still in critical years of both normal physiological and athletic development. This ignorance is what can lead to the premature end of many young athletes’ careers. As a collegiate runner who at one point ran 70+ miles per week, weighed under 100 pounds and suffered from five stress fractures within a 5-year period, the first author knows this uninformed pattern all too well. Fortunately, this ignorance can be corrected with the correct information obtained through well-designed studies that include female athletes

While the research is still trailing behind, there are coaches that have succeeded in catering to their female athletes’ specific needs. We have seen some tremendous accomplishments by both collegiate and professional female runners in the last few years, and we believe that there are a small number of coaches out there who “get it”. We have no doubt seen that these coaches have utilized the latest available performance research and that it has, in part, helped with the success of their athletes. What we think these coaches do, that many researchers still do not, is consider the factors that make these athletes different from their male counterparts and embrace them—whether scientific or not. Many scientists still see sex differences and hormonal fluctuations in women as burdens to their research. If progress is to be made, we can no longer see women’s differences as a burden, and instead, we need to welcome them in research and have advocates in all areas of science and funding agencies.

## 7. Conclusions

We have only just scratched the surface in the exploration of sex differences in endurance-exercise performance, including factors contributing to VO_2max_, other physiological determinants of performance, and sex differences in training strategies. Before we can definitively answer the question of whether or not women are capable of matching the performance of men, we must gain a better understanding of how women uniquely respond and adapt to different forms of endurance training. Along with the differences highlighted above, women exhibit physiological changes with the fluctuation of hormones throughout the menstrual cycle, have unique biomechanical profiles, and experience different lifestyle obstacles when it comes to endurance training. Thus, females likely require different nutritional, recovery, and injury prevention strategies than male athletes to reach optimal performance [115]. While in recent years, more female participants have been included in sports and performance research than in previous years, these mostly include recreational athletes. High-performance females are further underrepresented in the sports performance literature, limiting the ability to maximize the performance potential of these athletes [115]. Women have and will continue to break barriers in endurance-exercise performance; it is time to put these athletes at the forefront of sports performance research.
